# Inconsistent phylogeographic pattern between a sperm dependent fish and its host: *in situ* hybridization *vs* dispersal

**DOI:** 10.1186/s12862-016-0754-5

**Published:** 2016-09-06

**Authors:** Roland Vergilino, Christelle Leung, Bernard Angers

**Affiliations:** Department of biological sciences, Université de Montréal, C.P. 6128, succ. Centre-ville, Montréal, Québec H3C 3J7 Canada

**Keywords:** Local hybridization events, Gynogenesis, *Chrosomus eos-neogaeus*, Population genetics, Spatial autocorrelation, Sperm-dependence, Host-parasite

## Abstract

**Background:**

Co-dispersal of sperm-dependent hybrids and their sexual relatives is expected to result in consistent spatial patterns between assemblages of hybrids and genetic structure of parental species. However, local hybridization events may blur this signal as assemblages could be organized under different connectivity constraints. This study aims at testing the hypothesis of local hybridization events by comparing the assemblage of hybrid fish *Chrosomus eos-neogaeus* to the genetic diversity of one of its parental species, *Chrosomus eos*.

**Results:**

An extensive survey performed on a total of 132 sites located in two regions of Southern Quebec (West-Qc and East-Qc) revealed a distinct organization of hybrid lineages. One of the six hybrid lineages detected in West-Qc is widespread throughout this region resulting in a low α-diversity (1.38) and β-diversity (4.35). On the other hand, 36 hybrid lineages were detected in East-Qc and displayed narrow geographic distributions leading to a high α-diversity (2.30) and β-diversity (15.68). In addition, the *C. eos* multilocus haplotype of several of these hybrids is assigned to their respective sympatric *C. eos* population. Finally, contrasting with hybrids, the paternal species *C. eos* displayed a higher ρ_ST_ in West-Qc (0.2300) than in East-Qc (0.0734).

**Conclusion:**

The unusually high diversity of hybrid lineages in East-Qc as well as the spatial organization and the close genetic relationship with *C. eos* sympatric populations support the hypothesis that multiple hybridization events occurred *in situ*. These findings coupled to the near absence of the maternal species *Chrosomus neogeaus* suggest that the decline of this species could be the trigger event at the origin of the high rates of spontaneous hybridization in this region.

**Electronic supplementary material:**

The online version of this article (doi:10.1186/s12862-016-0754-5) contains supplementary material, which is available to authorized users.

## Background

If contemporary evolutionary forces and current landscape elements contribute to the spatial organization of genetic diversity [1–6], historical factors such as geological events, glacial cycles and post-glacial dispersal are also known to play a major role in shaping current species distributions [7–10]. Because all these factors act independently on species populations, two unrelated species are not expected to display a similar genetic structure except if they have been equally influenced by the action of important geological processes (e.g. vicariance [11–13]) or are linked by strong ecological associations (e.g. host–parasite [14–17]).

In Metazoans, organisms resulting from interspecific hybridization are often associated with asexual reproduction and lead to the formation of all-female unisexual lineages [18–22]. Gynogenesis is the most frequent mode of reproduction observed in unisexual vertebrates [23–25] and is present in fishes and amphibians [25, 26]. Clonal reproduction could result in the competitive exclusion of the parental species population by the asexual hybrids [27, 28] benefiting from demographic advantages (the so-called "Two-fold cost of sex" [29]). In addition, asexual hybrids avoid outbreeding depression by perpetuating the F1 generation and the benefits of heterosis [30]. However, gynogenesis, also called sperm-dependent parthenogenesis, requires the sperm of either parental species to trigger embryogenesis [31]. Sperm-dependent unisexual organisms are therefore closely tied to parental species for their persistence and are restricted to the geographic range of their sexual host [32].

Hybrids and their ecological relationships with parental species are of great importance to the study of evolution [18, 33–36] and conservation biology [28, 37, 38]. Hybridization also plays a substantial role in ecological differentiation [39, 40] and species diversification [18, 41–43]. Studying the evolution of gynogenetic organisms is key in order to assess how genomes without recombination deal with genetic decay and what ecological and behavioural processes may create and maintain stable coexistences between sexual and sperm-dependent unisexual organisms [31]. Elucidating when and how often gynogenetic hybrid clones originate is particularly important as a prerequisite to approach these topics. However, uncovering the origin of hybrids often represents a challenging task [44–47].

This study aims at assessing the origin of sperm-dependent hybrid lineages by comparing the distribution of hybrids with one of their parental species. We used fishes from the *Chrosomus eos-neogaeus* complex (Cyprinidae; Teleostei) that includes paternal species northern redbelly dace *Chrosomus eos*, maternal species finescale dace *Chrosomus neogaeus* and gynogenetic hybrids *Chrosomus eos-neogaeus* [48, 49]. While these hybrids are widely distributed in North-America, previous studies reported a low number of hybrid lineages and the presence of the same lineage in different hydrographic networks, suggesting few hybridization events predating the end of the Pleistocene [50, 51]. However, the presence of distinct hybrid lineages within a drainage basin in the Eastern region of Quebec (Canada) raises new questions on the origin and diversity of hybrid lineages [50].

In freshwater environments formerly covered by glaciers, the period during which hybridization events occurred relative to the Pleistocene-Holocene boundary strongly shaped current patterns of diversity at the regional scale [50]. Given the ecological dependence of gynogenetic hybrids to their parental species, different predictions could be formulated depending on whether hybridization events occurred in glacial refuges, during the Pleistocene, or *in situ,* during the Holocene.

Hybrids produced during the Pleistocene may have benefited from the pro-glacial lakes and temporary bridges among hydrographic networks [6, 8, 13, 52, 53] to spread throughout drainage basin(s) of one particular region [50]. This would have resulted in homogeneity in the initial assemblage of hybrids across drainage basins. Following colonization, isolation of hydrographic networks and the process of lineage sorting may have resulted in different patterns according to the number of lineages in the founder group. Whereas the dispersal of a single lineage would result in a homogeneous distribution of the same genotype across drainage basins [50, 53–55], colonization by multiple lineages would provide a patchwork of assemblages with narrower geographic distribution and lineages present in multiple assemblages. On the other hand, hybridization events may have occurred during the Holocene in conditions similar to the current landscape where each hydrographic network is isolated from each other. These assemblages of hybrids are expected to have a narrow geographic distribution [56] but should harbor assemblage-specific lineages even in the absence of corresponding genetic structure in parental species. Both colonization by multiple lineages and local hybridization events are expected to produce a pattern of spatial autocorrelation limited by very short distances for the distribution of hybrid lineages. However, these scenarios can be discriminated, as only the multilocus genotypes from *in situ* hybridization events are expected to match the genetic diversity in the sympatric parental populations.

In this study, we report the results of an extensive survey of 132 sites from two regions (West-Qc and East-Qc) of Southern Quebec (Canada). We determined the diversity of hybrid lineages and the genetic diversity of parental species. Spatial autocorrelation analysis and assignment tests were performed to assess the hypothesis that hybrids were produced *in situ.*

## Methods

### Sampling and identification

**Fig. 1:**
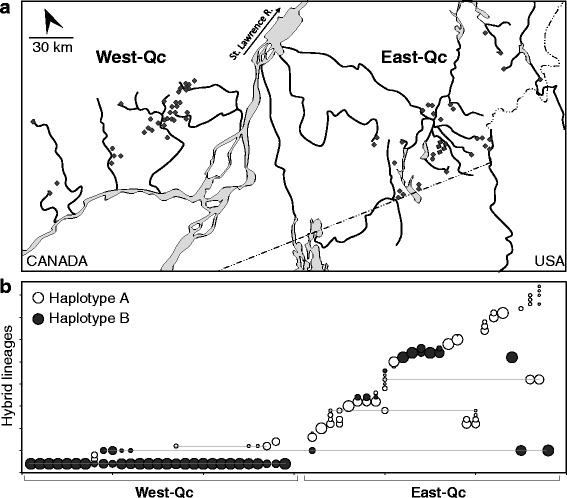
Distribution of individuals from *C. eos-neogaeus* complex. **a** Map of Southern Quebec (Canada) where *C. eos*, *C. neogaeus* and/or hybrid *C. eos-neogaeus* individuals were detected (refer to Additional file 1 for the complete list of sampled sites). **b** Lineage distribution in West- and East-Qc. Sites follow the same order than Additional file 1; circle areas refer to the relative abundance of lineages within a given site; horizontal lines connect lineages that are found in sites distant by more than 7 km

A total of 132 sites were sampled throughout two regions in Southern Quebec (Canada) including 63 sites in the Laurentians region (West-Qc) and 69 sites in the Eastern Townships (East-Qc) that span areas of approximatively 3,800 km^2^ and 4,000 km^2^ respectively (Fig. 1a, Additional file 1). Sampled individuals were identified visually as *Chrosomus sp.* according to external morphological characteristics [57]. Morphological identification was confirmed using genetic markers. DNA was extracted from the upper lobe of the caudal fin and genetic identification of the different biotypes of the *C. eos-neogaeus* complex was performed according to Binet and Angers [58].

### Analysis of hybrid lineages

For each individual, a multilocus genotype was defined using the size variation of an intron of the PEG1/MEST gene as well as six microsatellite loci (Pho-1, Pho-2, Pho-60, Pho-61, Ca-12 and Seat-412 [50, 58, 59]) amplified according to Angers and Schlosser [50]. Pho-1, Pho-2, Pho-60, Pho-61 are specific to the *C. eos* genome while PEG1/MEST, Ca-12 and Seat-412 amplify both *C. eos* and *C. neogeaus* genomes leading to a total of ten nuclear loci for hybrid genotypes. *Chrosomus eos* and *C. neogeaus* alleles of hybrid genotypes could be distinguished for PEG1/MEST and Ca-12 loci, but not for Seat-412 for which allelic size ranges overlap between parental species. This locus was then excluded from the analyses that required *C. eos* genotypes.

A hybrid lineage is defined as all individuals originating from a given hybridization event [50]. In the absence of recombination and segregation, individuals of a given lineage were thus expected to display the same multilocus genotype. However, variants could be detected due to the high mutation rate of microsatellite loci. They generally differ by a single mutation at one or a few loci [50]. A given lineage is therefore characterized by a consensus genotype defined by the allele of invariant loci and the more abundant allele for the variable loci.

Triploid hybrids are abundant in the *Chrosomus eos-neogaeus* complex [49]. They are exclusively produced *de novo* following the incorporation of the spermatozoid genome into a diploid hybrid egg [48]. Since genotypes of triploid individuals include the consensus multilocus genotype of a given hybrid lineage in addition to the spermatozoid haplome, it is possible to unambiguously identify the lineage from which triploid individuals derived. In the absence of a match between a triploid genotype and consensus lineages, this genotype is considered as an additional lineage. However, it becomes impossible to discriminate the *C. eos* multilocus hybrid haplotype from the spermatozoid haplome in lineages represented by a single triploid individual.

The hybrid lineages were further characterized at the mitochondrial level using a fragment of the cytochrome oxidase I (COI) gene to test the hypothesis of a unique *vs* multiple hybridization events. Amplifications were carried out using 5’-CCAGTGTTAGCAGCCGGAAT and 5’-GGGTGTCTACGTCTATGCC primers. PCR amplicons were differentiated using the SSCP (Single-Stranded Conformation Polymorphism) method [60] and variants were sequenced.

We infer genetic relationships among hybrid genotypes to assess whether they are derived from single or multiple hybridization events. The clonal distance [61] was used to assess the number of pairwise differences over all microsatellite loci, except Seat-412, according to a stepwise mutation model (SMM). An unrooted Neighbor-Joining tree was constructed using the derived distance matrix. Assuming a SMM model, if the distinct hybrid lineages derived from a single clone, the unrooted tree should have a star-like conformation with short external branches.

Regional diversity of hybrid lineages was assessed by calculating α- and β-diversity. The degree of uniqueness in terms of their lineage composition was assessed with the Local Contribution to Beta Diversity (LCBD) index [62] for each site. LCBD values were computed using abundance-based Jaccard coefficient [63] and tested using 999 permutations to identify sites that contributed the most to the beta diversity index throughout the sampling area [62].

Mantel correlograms were also constructed to establish the spatial distribution of distinct hybrid lineages for each region (East-Qc and West-Qc). This analysis was used to determine whether sites closer geographically displayed higher similarity in their lineage composition [64, 65]. Historical processes are not be well characterized by elements of the current landscape [66], especially when the sampling sites are located in the headwaters of distinct hydrographic networks. Moreover, temporal interconnections may exist between two sites from distinct hydrographic networks [66, 67]; we therefore used straight line distance between sites in addition to waterway distance. Binary (presence/absence) dissimilarity values among sites were used as a dependent variable and geographic distance matrix as an independent variable. Number of distance classes were determined according to Sturges’ rule and Mantel statistics were tested with 999 permutation using the correction for multiple tests proposed by Holm [68]. Dissimilarity calculations, Mantel correlograms, and permutations tests were performed using the *vegan* package [69] for the R 3.2.2 software.

### Analysis of parental species

Populations of parental species *C. eos* were analyzed using the same nuclear markers than those used for hybrids. For each population, 7–25 individuals were genotyped (Additional file 2). Allele frequencies for each locus and genetic diversity (*H*_*E*_) were calculated using FSTAT v.2.9.3 [70]. Linkage disequilibrium between locus and departure from Hardy-Weinberg equilibrium was tested for each and all nuclear loci for each population and significance was assessed by 10,000 iterations using GENEPOP v.4.2 [71, 72]. Sequential Bonferroni corrections were applied to all multiple comparisons [73].

A Principal Component Analysis (PCA), as implemented in the *adegenet* package [74, 75] for the R 3.2.2 software was realised to assess the organization of the genetic diversity among *C. eos* populations. To infer population differentiation, allelic differentiation over all loci and between populations was quantified by computing Weir and Cockerham’s [76] estimator of pairwise F_ST_(θ) based only on the allelic diversity and Michalakis and Excoffier’s [77] estimator of pairwise Rho_ST_(ρ_ST_) taking into account allelic size using ARLEQUIN v. 5 [78]. Significance of pairwise differences was assessed by 1,000 permutations. The comparison of F_ST_ and ρ_ST_, which behave differently with regard to mutation, allows to make historical and demographical inferences on population differentiation processes [79, 80]. We estimated pRho_ST_ (ρ_ST_ computed after allele size permutation, [80]) to test if the mutation process has contributed to population differentiation using the allele size permutation test with 20,000 randomization assuming that the loci analyzed follows an SMM-like model as implemented in SpaGeDi v.1.5 [81]. A hierarchical analysis of genetic variation (AMOVA) implemented in ARLEQUIN v. 5 [78] was applied using both F_ST_ and ρ_ST_ to quantify the amount of genetic variance explained by the subdivision in regions (West-Qc and East-Qc) over the total genetic variance. Mantel correlograms were also constructed for *C. eos* populations from each region (East-Qc and West-Qc) by using Cavalli-Sforza and Edward’s D_CE_ genetic distance [82, 83] as a function of straight line or waterway distances.

### Assignment of the hybrid lineages

Assignments of hybrid lineages to *C. eos* populations was performed using all *C. eos* nuclear loci, except Seat-412. The likelihood that the *C. eos* multilocus haplotype of hybrid lineages originated from a given *C. eos* population was computed using the method based on allele frequencies [84]. The frequency of missing alleles was set at 10^−5^ and the probability was estimated by simulating 10,000 individuals based on the algorithm by Cornuet et al. [85], using the method implemented in Geneclass2 [86]. A percentage score for each population was calculated by dividing the assignment likelihood of each population to the sum of likelihoods across all populations tested.

## Results

### Distribution of biotypes

Members of the *Chrosomus eos-neogaeus* complex (*C. eos*, *C. neogaeus* and/or hybrids) were captured in 70 of the 132 sampled sites, more specifically in 36 (57.14%) sites in West-Qc and 34 (49.28%) sites in East-Qc. The maternal species *Chrosomus neogaeus* is nearly absent from both regions as successful captures of this species were restricted to three sites (AS-3, SF-12 and CH-1, Additional file 1). Because *C. neogaeus* has been sampled at multiple occasions at sites AS-3 and SF-12, we can exclude particular behaviours or habitat specificity that could hamper its capture and assume a very low abundance of this species through the sampled area. The paternal species *C. eos* and hybrid individuals were respectively sampled in 27 and 29 sites in West-Qc and 24 and 27 sites in East-Qc (Fig. 1, Additional file 1).

The paternal species *C. eos* and hybrid individuals were sympatric in 37 sites (20 in West-Qc and 17 in East-Qc). A total of 14 sites (7 in each region) contained exclusively *C. eos* individuals. Hybrids were detected in absence of parental species in 18 sites (9 in each region). However, this should be interpreted with caution as the low number of captures at those sites (<6 individuals) reduces the probability of detecting parental species.

### Diversity of hybrid lineages

The genetic survey of hybrid individuals revealed a high diversity of genotypes that could be clustered in 41 distinct consensus genotypes. As observed in previous studies [50], several genotypes differed from a consensus by a single or a few mutations (Additional files 1 and 3). Two distinct mitochondrial haplotypes are detected over all hybrid lineages. Sequencing allowed recovery of the A and B haplotypes found by Angers and Schlosser [50] (NCBI accession numbers [EU014286] and [EU014287] respectively). These haplotypes differ by a single bp out of 685 bp which corresponds to a divergence of 0.15%. Haplotypes A and B are associated to 34 and 7 distinct hybrid genotypes respectively (Fig. 1b, Additional file 1).

**Fig. 2:**
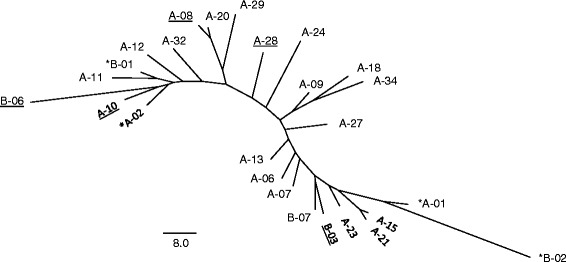
Relationships among hybrid lineages. Neighbor-Joining tree inferred from clonal distances calculated from microsatellite loci among the consensus genotypes of 26 hybrid lineages. Lineages are identified according to mitochondrial haplotypes (A or B), regions (asterisk refers to West-Qc) and *C. eos* alleles at PEG1/MEST locus (underlined or not)

Of the 41 hybrid lineages, 15 are represented by a single triploid individual (Additional file 3) for which it was impossible to characterize the hybrid genotype. Analysis of the remaining 26 hybrid multilocus genotypes specific to the paternal *C. eos* species (Additional file 3) revealed a high allelic diversity, ranging from 9 (Ca-12) to 15 (Pho-1 and Pho2) alleles. This represents an average of 10.5 alleles/loci and a Nei’s gene diversity ranging from 0.815 (Ca-12) to 0.954 (Pho1), values that are similar to the ones measured within *C. eos* populations. The Neighbor-Joining tree of the multilocus genotypes (6 loci) of these 26 hybrid lineages shows long internal branches and does not present a star-like topology (Fig. 2). Lineages did not cluster according to regions, mitochondrial haplotypes or alleles at the intron of the PEG1/MEST gene. There was an average of 50.65 (± 21.73) stepwise mutations among consensus genotypes and up to 122 mutations between the most distant genotypes. On the other hand, the average number of pairwise differences within-lineage estimated on all lineages with more than a single individual was 3.65 (± 2.50) stepwise mutations. In addition, variants of the mitochondrial and PEG1/MEST loci appeared to segregate independently and this topology requires four homoplasic mutations occurring on each of these markers. Altogether, this confirms that the diversity of hybrids is not the result of the diversification of one (or few) clone(s) via mutation process but originated from distinct hybridization events.

**Fig. 3:**
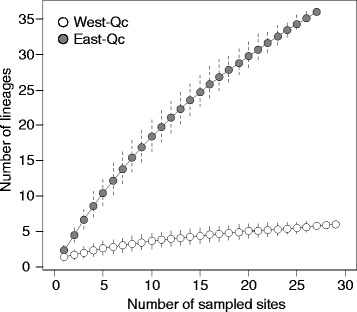
Lineages richness of West-Qc and East-Qc. Mean lineages accumulation curve according to sampled sites and standard deviation were estimated according to random permutations without replacement [95]

The diversity of hybrid lineages is highly different between the two regions: sites in West-Qc (0.0083 ≤ LCBD index ≤ 0.0192) are less unique and diverse than those in East-Qc (0.0243 ≤ LCBD index ≤ 0.0269) and only sites from East-Qc contribute significantly (LCBD, *P* < 0.05) to the total β-diversity. Only 6 lineages were detected in West-Qc; 1 lineage is widespread and was detected in all of the 29 sampling sites containing hybrids. Other lineages are limited to a single site or a few geographically close sites (Figs. 1 and 3). This leads to a very low α-diversity (1.38) as well as β-diversity (4.35). On the other hand, 36 lineages were detected in the 27 sampling sites containing hybrids in East-Qc (Figs. 1 and 3). These lineages displayed a narrow geographic distribution and up to six lineages were found in sympatry. This leads to a higher α-diversity (2.30) and β-diversity (15.68) than observed in West-Qc. The geographically close sites harbouring similar hybrid diversity could be grouped into 15 assemblages of hybrid. Only 3 lineages are detected in distinct assemblages (A-09; A-15; B-02). Interestingly, the B-02 hybrid lineage is found in both West-Qc (sites AS-12 and AS-13; AS-14; AS-15; AS-16) and East-Qc (sites YA-1, SF-19, and SF-22) (Additional file 1).

**Fig. 4:**
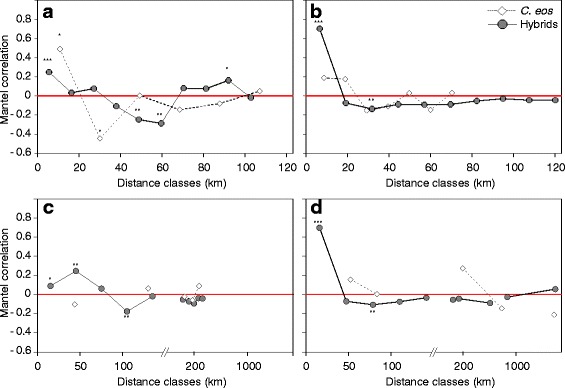
Mantel correlograms of the distribution of the hybrid lineages and *C. eos*. Jaccard distance and Cavalli-Sforza and Edward’s D_CE_ genetic distance were used to assess the differences among sites respectively for hybrid lineages and *C. eos* populations. Straight line distance in (**a**) West-Qc and (**b**) East-Qc and waterway distance in (**c**) West-Qc and (**d**) East-Qc. Stars indicate significant autocorrelation values: ***, *P* < 0.001; **, *P* < 0.01; *, *P* < 0.05 after Holm corrections

The assemblage of hybrids is similar among geographically close sites (e.g. within a given minor drainage), but are completely different as distance increases in East-Qc. Therefore, strong and significant positive spatial autocorrelations were observed for the first distance class using straight as well as waterway distances (Fig. 4), indicating that similar lineages are observed in close sites (< 7 km for straight line distance, 15 km for waterway distance) compared to more distant sites.

Significant positive autocorrelations are also detected in West-Qc for the first distance classes ((< 7 km for straight line distance, 16 and 47 km for waterway distance) Fig. 4). The difference observed between straight line and waterway distances suggests the presence of historical and/or contemporary interconnections between drainages for the West-Qc.

The correlation value is significantly higher for straight line distance in East-Qc (r = 0.703) compared to West-Qc (r = 0.248) (Fisher’s z = 4.1918, *P* < 0.0001), as well as for waterway distance in East-Qc (r = 0.6977) compared to West-Qc (first distance class: r = 0.0901; Fisher’s z = 4.1119, *P* < 0.0001; second distance class: r = 0.2463, Fisher’s z = 2.8310, *P* = 0.0023 [87]).

### Characterization of *Chrosomus eos* populations

*Chrosomus eos* populations were characterized according to 16 sites for which more than 7 individuals were sampled. High allelic diversity is observed for those populations, with the number of alleles per locus ranging from 25 (Ca-12) to 32 (Pho-1). An average of 10.76 alleles/locus/site and 0.88 for Nei’s gene diversity (H_E_) are observed (Table 1). The number of alleles and Nei’s gene diversity are not significantly different between regions (*P* > 0.240).

**Table 1:** Genetic diversity in hybrid *Chrosomus eos*-*neogaeus* and in the sexual species *Chrosomus eos* between West-Qc and East-Qc regions

Hybrids *C. eos-neogaeus*					
	N sites (n)	N lineages	α-diversity	β-diversity	
West-Qc	29 (346)	6	1.38	2.30	
East-Qc	27 (340)	36	4.35	15.68	
Total	56 (686)	41	1.82	22.51	
*Chrosomus eos* populations					
	N sites (n)	k	H_S_	F_ST_	ρ_ST_
West-Qc	7 (132)	23.33	0.8653	0.0740	0.2302
East-Qc	9 (118)	25.27	0.8913	0.0327	0.0734
Total	16 (250)	28.33	0.8799	0.0579	0.1663

Number of sites and total number of individuals analysed is provided. For hybrids, diversity is represented by the number of lineages as well as α- and β-diversity. Diversity of the sexual species is given in number of alleles (k), mean of Nei’s gene diversity, F_ST_ and ρ_ST_

No significant linkage disequilibrium between loci has been detected (Fisher's method, *P* > 0.937). Global H-W test revealed four populations from West-Qc (AS-5, AS-17, BA-1, RO-1) showing a significant deviation from H-W expectations (using sequential Bonferroni corrections). This is mainly due to a deficiency of heterozygotes at the microsatellite locus Pho-2. When this locus is excluded from the analysis, no further significant deviation from H-W expectations is detected.

**Fig. 5:**
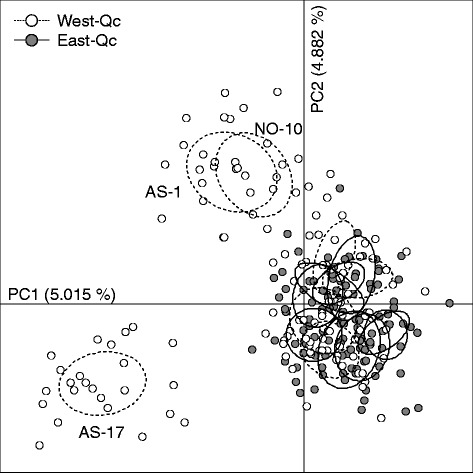
Results of principal component analysis of *C. eos* population genetics. Open and black circles refer to populations of West-Qc and East-Qc respectively. Only the most divergent populations are identified

Populations are genetically different as only 15 of the 120 pairwise comparisons were not significant (using sequential Bonferroni corrections). All of them involved populations from East-Qc. The PCA analysis revealed a very important overlap among all populations (Fig. 5), as a result of a low global F_ST_ (0.0579) and ρ_ST_ (0.1663). Three populations from West-Qc (AS-17, AS-1, NO-10; Fig. 5) are different from the rest. The difference of F_ST_ between regions (West-Qc: 0.0739; East-Qc: 0.0327) is not significant (*P* = 0.180) but allele size permutation tests performed on ρ_ST_ (West-Qc: 0.2300; East-Qc: 0.0734) provides a significant result (*P* < 0.0005).

The lack of spatial patterning between regions is supported by hierarchical analyses of genetic variation (AMOVA) using either allele diversity (F_CT_ = 0.00291, *P* = 0.23949) or allele size diversity (ρ_CT_ = 0.01620, *P* = 0.23656). Moreover, Mantel tests revealed no significant isolation-by-distance patterns, neither for the whole area (R^2^ = 0.0137, *P* = 0.132) nor within each region (West-Qc: R^2^ = 0.0284, *P* = 0.266; East-Qc: R^2^ = 0.0337, *P* = 0.146). A significant positive spatial autocorrelation signal is detected in the first distance class in West-Qc but only for straight line distance (Fig. 4a, c). This result is mostly due to the populations AS-1 and NO-10 that are genetically similar one to each other but highly divergent from the other populations; both populations are geographically close but in distinct hydrographic networks (Figs. 4a and 5). On the other hand, no significant spatial autocorrelation was observed for the East-Qc populations (Fig. 4b, d). This result reflects a uniform distribution of alleles in *C. eos* populations, that strongly contrasts with the distribution of hybrid assemblages.

### Assignment of hybrid lineages to *Chrosomus eos* populations

Because assignment tests are sensitive to sample size, sites geographically close and harboring the same assemblage of hybrids were pooled together. A total of 18 hybrid lineages are found at these sites (Table 2). However, 3 of them are represented by a single triploid individual and were excluded from this analysis because of our incapacity to infer the *C. eos* haplotype (Additional file 3). Ten of the 12 lineages exclusive to East-Qc are assigned with a high probability to their respective sympatric *C. eos* population and 9 of them with the highest score over all sites (Table 2). Lineage A-01 exclusive to West-Qc is assigned with the higher score to East-Qc populations while lineage B-01 is assigned to one of the three sympatric *C. eos* populations with the highest score. The B-02 lineage detected in both regions is assigned to its sympatric *C. eos* population in East-Qc but not in West-Qc.

**Table 2:**
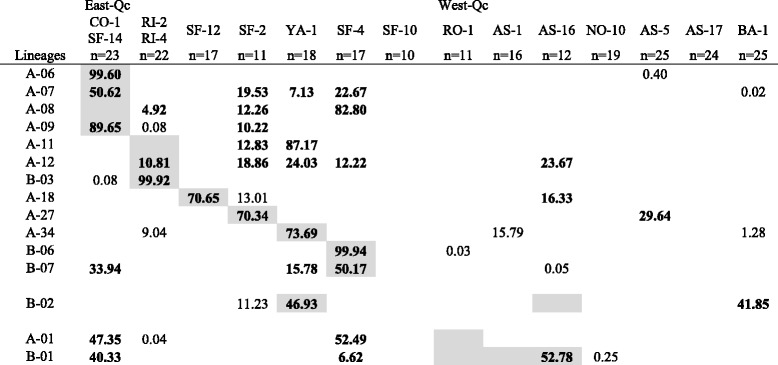
Results of the assignment of hybrid lineages to the *Chrosomus eos* populations

Assignment score of the *C. eos* genotype for the 15 hybrid lineages detected to a given *C. eos* population (Additional file 1). Shaded indicated site(s) where hybrid lineage was detected. Bold number refers to a probability of 100 % that a given lineage belongs to a reference population

## Discussion

The joint analysis of *C. eos-neogaeus* hybrid diversity and the paternal species *C. eos* populations genetic diversity in two regions of southern Quebec revealed two striking results. First, a highly distinct geographic organization of the assemblages of hybrids is observed between the two regions. Second, in spite of the sperm dependence of hybrids, there is a lack of correspondence between the spatial organization of hybrid lineages and the genetic diversity of paternal species *C eos*, this is especially clear in East-Qc.

### Uniform distribution of *Chrosomus eos*

The results revealed a lack of population structure for the *C. eos* species in the studied areas. Even the separation of both regions by the St-Lawrence River has a limited impact on the phylogeographic structure of *C. eos* populations as less than 2% of the total genetic variance is explained by the subdivision in regions. Drainage basins do not seem to explain population genetics structure of *C. eos* either. Although allele frequencies of most populations are significantly different, the differentiation is limited and only three populations from West-Qc are clearly distinct from the other populations. Such organization of the genetic diversity suggests that most of the *C. eos* populations likely dispersed through temporary hydrologic bridges from one single founding group after the ice-sheet retreated [13, 52].

### Contrasting patterns in hybrid diversity

The hybrids sampled in this study are highly diverse with 41 distinct hybrid lineages. The number of stepwise mutations among genotypes is 14 times larger than the average number of pairwise difference within lineage. In addition, phylogenetic relationships among hybrids revealed that lineages do not cluster according to variants of the mitochondrial and PEG1/MEST loci. These results rule out the probability of derived clones from a unique hybridization event and suggest hybrid lineages have been produced through distinct hybridization events (e.g. [88–90]). This high diversity contrasts with previous studies on *Chrosomus eos-neogaeus* reporting a single or a few lineages within a given region [48, 50, 51].

The striking difference in the distribution of hybrid lineage diversities between the two regions likely reflects distinct scenarios in the origin and colonization of hybrid lineages in West-Qc and East-Qc. In West-Qc, the widespread distribution of the B-01 hybrid lineage leads us to conclude, as previously stated by Angers and Schlosser [50], that this lineage occurred during the Pleistocene period and has colonized the sites of West-Qc via pro-glacial temporary drainages. This pattern has been observed previously, including in the *Chrosomus eos-neogaeus* complex [50, 51] and is common in sperm-dependent unisexual hybrid species [53–55].

The narrow distribution of the other lineages specific to West-Qc (A-02, A-03 and A-04) is however more difficult to interpret in a context of postglacial colonization. If a postglacial colonization appears unlikely, these hybrid lineages are good candidates for the hypothesis of locally produced hybrids in West-Qc. These lineages have a narrow distribution similar to the one observed in the assemblages of hybrids of East-Qc, they are always in sympatry with the B-01 lineage and two of these additional lineages occurred in the same site while most of the West-Qc sites harbored only a single lineage.

Contrasting with the homogeneity of West-Qc, 15 distinct assemblages of hybrids composed of 36 distinct lineages are observed in East-Qc. Except for three lineages that can be found in assemblages distant by more than 7 km using straight line distance (15 km using waterway distance), most assemblages harbor specific lineages and have a narrow distribution leading to highly significant positive spatial autocorrelation patterns. Assignment tests revealed a close genetic relationship between hybrids from East-Qc and sympatric *C. eos* populations as most of the hybrids tested were assigned to their respective *C. eos* population. In addition to the large diversity and the positive spatial autocorrelations detected in hybrids compared to the lack of organization in the genetic diversity of the paternal species *C. eos*, these results give weight to the hypothesis that multiple hybridization events between *C. eos* and *C. neogaeus* occurred locally in several sites in East-Qc. Given the high waterway distances among drainages, positive spatial autocorrelations detected at short distances using straight line as well as waterway distances also support the hypothesis that hybridization events occurred after disappearance of pro-glacial temporary bridges.

Post-glacial colonization alone cannot explain the observed distribution of hybrid lineages in both regions. The wide distribution of the B-01 lineage in West-Qc suggests a post-glacial colonization. However, hybridization events likely occurred *in situ*, in order to yield the current distribution of the alternative lineages. In East-Qc, the distribution of few lineages in different assemblages is likely the result of dispersal although numerous local hybridization events are responsible for the observed hybrid assemblages. *In situ* hybridization events appear to be rare in most sperm-dependent unisexual hybrids (but see [56]) and our results provide the first strong evidence of locally produced hybrid lineages during the Holocene in the *Chrosomus eos-neogaeus* system.

### Diversity and landscape elements

In spite of a higher number of sites sampled in this survey compared to the one performed by Angers and Schlosser (2007), no additional lineages was detected in the lakes of West-Qc. Interestingly, all of the additional hybrid lineages detected in this survey were found in small streams. This leads us to associate the difference in diversity of the assemblages of hybrids between regions to the different geomorphologic characteristics of their respective hydrographic networks. West-Qc is characterized by thousands of lakes and ponds whereas a dendritic system of ephemeral and permanent streams is observed in East-Qc; lakes and ponds are nearly absent from this last region.

The very low diversity of the West-Qc region may be explained by the presence of the widespread B-01 lineage. The high abundance of this lineage in most of the lakes could be related to extensive postglacial colonization. Its presence in most of the lakes may prevent the persistence of other hybrid lineages in lakes as expected under the neutral theory of biodiversity [91], or be the result of competitive exclusion of alternative lineages in lake environments.

### Demographic decline of *Chrosomus neogaeus*

The results of this survey are consistent with previous studies reporting low abundance of the maternal species *Chrosomus neogaeus* [50]. In addition, the presence of *C. eos-neogaeus × eos* as triploid hybrids, never *C. eos-neogaeus × neogaeus,* indirectly confirmed the near absence of *C. neogaeus* in both regions. Gynogenetic *C. eos-neogaeus* require the sperm of either parental species to reproduce, the spermatozoid genome is occasionally incorporated into the diploid hybrid egg leading to the formation of triploid hybrids. These triploid hybrids are observed in high proportion in every hybrid populations but are only produced *de novo* [48]. The presence of an additional *C. eos* genome in triploid individuals indicates that hybrids use the sperm of the *C. eos* species, not *C. neogaeus* to trigger the development of their eggs.

The reasons for the scarcity of the *C. neogaeus* species in these regions remains to be investigated, however, multiple local hybridization events may be indicative. According to the Hubbs principle, hybridization is more likely to occur if one of the sympatric species becomes rare [92]. Hybridization events involving now extinct species have been reported in multiple species including fishes [93, 94]. Environmental changes following the end of the Pleistocene may have triggered the demographic decline of *C. neogaeus* populations, increasing the probability of interspecific mating by *C. neogaeus* females. Production of gynogenetic unisexual hybrids may have accelerated the disappearance of *C. neogaeus* populations by outcompeting them due to the alleviation of the “double cost of sex” in hybrids. While this scenario is hypothetical, it provides an explanation to the high diversity of hybrids as well as to the near lack of maternal species.

## Conclusions

This study supported the hypothesis of multiple hybridization events between *Chrosomus eos and C. neogaeus* occurring during the Holocene in East-Qc. This explains the difference in the diversity and distribution of hybrid lineages between regions separated by the St Lawrence River in absence of marked differences among *C. eos* populations. This highlights distinct historical processes that act in shaping the diversity in the assemblage of hybrids in each region. This unusually high diversity of hybrid lineages in East-Qc coupled to the near absence of the maternal species *Chrosomus neogeaus* suggests that the decline of this species could be the trigger event at the origin of the high rates of spontaneous hybridization in this region.

## Additional files

Additional file 1: Characteristics of the different sampled sites. List of sampled sites with their geographical coordinates and biotypes composition. E, N and H respectively refer to *Chrosomus eos*, *C. neogaeus* and the hybrids. Hybrid lineages of each site are identified. (PDF 73 kb)
Additional file 2: Multiloci genotype of the parental species *C. eos* individuals. Nuclear genotype of every *C. eos* individuals used to assess the paternal species genetic diversity and structure. For each individual, region and sampled sites are provided. (PDF 348 kb)
Additional file 3: Consensus nuclear genotype and mitochondrial haplotype of the different lineages. Nuclear genotype and mitochondrial haplotype of the different lineages. Allele size refers to the more common genotype and bold characters to variable loci within a given lineage. (PDF 46 kb)
