# Supplementary material for: Inconsistent phylogeographic pattern between a sperm dependent fish and its host: in situ hybridization vs dispersal
Source: BMC Evol Biol. 2016 Sep 6;16:183. doi: 10.1186/s12862-016-0754-5 (PMC5012089; doi:10.1186/s12862-016-0754-5)
Supplement: Additional file 1: — Characteristics of the different sampled sites. List of sampled sites with their geographical coordinates and biotypes composition. E, N and H respectively refer to Chrosomus eos, C. neogaeus and the hybrids. Hybrid lineages of each site are identified. (PDF 73 kb) [file 12862_2016_754_MOESM1_ESM.pdf]

Additional file 1. Characteristics of the different sampled sites. Biotypes E, N and H respectively refer to *Chrosomus eos, C. neogaeus* and the hybrids.

Region	Drainage Bassin	Sites	Latitude	Longitude	Biotype	No. of hybrid lineages	Names of hybrid lineages
West-Qc	Assomption	AS-1	N 45° 55' 00.9"	W 74° 04' 22.5"	E, H	1	B-01
		AS-2	N 45° 54' 29.4"	W 74° 00' 20.7"	H	1	B-01
		AS-3	N 45° 54' 53.3"	W 74° 01' 40.9"	E, H, N	1	B-01
		AS-4	N 45° 58' 59.2"	W 74° 01' 06.0"	E, H	1	B-01
		AS-5	N 45° 59' 17.1"	W 74° 00' 24.7"	E	-	-
		AS-6	N 45° 59' 22.7"	W 74° 00' 18.4"	E, H	1	B-01
		AS-7	N 45° 59' 23.5"	W 74° 00' 00.0"	E, H	1	B-01
		AS-8	N 46° 05' 20.8"	W 73° 57' 21.8"	E, H	1	B-01
		AS-9	N 45° 57' 09.9"	W 73° 56' 13.2"	E	-	-
		AS-10	N 46° 06' 01.7"	W 73° 55' 50.8"	H	1	B-01
		AS-11	N 45° 55' 10.7"	W 73° 55' 09.3"	E, H	3	A-02; A-03; B-01
		AS-12	N 46° 04' 39.4"	W 73° 53' 02.5"	H	2	B-01; B-02
		AS-13	N 46° 05' 13.6"	W 73° 52' 44.0"	E, H	2	B-01; B-02
		AS-14	N 46° 05' 32.3"	W 73° 52' 26.5"	E, H	2	B-01; B-02
		AS-15	N 46° 05' 34.6"	W 73° 52' 20.4"	E, H	2	B-01; B-02
		AS-16	N 46° 05' 35.3"	W 73° 52' 15.5"	E, H	1	B-01
		AS-17	N 46° 05' 32.3"	W 73° 48' 33.0"	E	-	-
			N 45° 56' 18.8"	W 74° 02' 08.1"	-	-	-
			N 45° 56' 59.0"	W 73° 59' 23.1"	-	-	-
			N 46° 03' 36.9"	W 73° 57' 21.0"	-	-	-
			N 45° 51' 55.9"	W 73° 57' 02.2"	-	-	-
			N 45° 51' 51.4"	W 73° 56' 39.2"	-	-	-
			N 45° 51' 48.8"	W 73° 55' 48.7"	-	-	-
			N 46° 04' 06.7"	W 73° 55' 40.2"	-	-	-
			N 46° 03' 30.2"	W 73° 55' 37.9"	-	-	-
			N 45° 54' 37.2"	W 73° 54' 15.3"	-	-	-
			N 46° 04' 56.6"	W 73° 52' 50.5"	-	-	-
			N 46° 02' 20.8"	W 73° 48' 27.4"	-	-	-
	Blanche	BA-1	N 45° 47' 08.1"	W 75° 14' 57.4"	E	-	-
	Du Nord	NO-1	N 46° 07' 51.7"	W 74° 26' 46.2"	E, H	1	B-01
		NO-2	N 45° 53' 41.9"	W 74° 21' 40.7"	E, H	1	B-01
		NO-3	N 45° 54' 17.9"	W 74° 19' 17.7"	E, H	1	B-01
		NO-4	N 45° 54' 16.3"	W 74° 18' 46.0"	E, H	2	A-01; B-01
		NO-5	N 45° 59' 38.0"	W 74° 18' 21.0"	H	1	B-01
		NO-6	N 45° 57' 58.3"	W 74° 01' 43.5"	E, H	1	B-01
		NO-7	N 45° 56' 37.6"	W 74° 11' 37.7"	E, H	1	B-01
		NO-8	N 45° 52' 31.4"	W 74° 08' 44.6"	E	1	B-01
		NO-9	N 45° 56' 04.3"	W 74° 06' 11.3"	H	1	B-01
		NO-10	N 45° 55' 32.4"	W 74° 03' 51.1"	E	-	-
		NO-11	N 46° 02' 16.0"	W 74° 03' 16.0"	E, H	1	B-01
		NO-12	N 45° 57' 35.9"	W 74° 03' 13.8"	H	1	B-01
			N 45° 54' 43.6"	W 74° 21' 13.0"	-	-	-
			N 45° 54' 36.9"	W 74° 21' 07.8"	-	-	-
			N 45° 54' 54.1"	W 74° 20' 45.5"	-	-	-
			N 45° 54' 11.8"	W 74° 18' 54.3"	-	-	-
			N 45° 55' 59.5"	W 74° 09' 15.6"	-	-	-
			N 45° 55' 56.0"	W 74° 09' 12.6"	-	-	-
			N 45° 55' 55.3"	W 74° 09' 11.0"	-	-	-
			N 45° 55' 58.0"	W 74° 08' 44.1"	-	-	-
			N 45° 50' 52.0"	W 74° 06' 53.3"	-	-	-
			N 45° 50' 11.7"	W 74° 05' 51.9"	-	-	-

Region	Drainage Bassin	Sites	Latitude	Longitude	Biotype	No. of hybrid lineages	Names of hybrid lineages
			N 45° 55' 56.0"	W 74° 04' 36.1"	-	-	-
			N 45° 57' 43.1"	W 74° 03' 03.7"	-	-	-
			N 45° 54' 30.5"	W 74° 00' 36.5"	-	-	-
	Petite Nation	PN-1	N 46° 12' 43.0"	W 75° 13' 60.0"	E	-	-
	Rouge	RO-1	N 45° 46' 38.9"	W 74° 34' 25.8"	E, H	2	A-01; B-01
		RO-2	N 45° 51' 44.0"	W 74° 33' 55.5"	H	2	A-01; B-01
		RO-3	N 45° 50' 42.6"	W 74° 33' 24.2"	E, H	2	A-01; B-01
		RO-4	N 45° 49' 44.0"	W 74° 31' 53.2"	H	2	A-04; B-01
			N 45° 49' 45.0"	W 74° 30' 52.5"	-	-	-
			N 45° 49' 49.8"	W 74° 30' 43.2"	-	-	-
			N 45° 49' 43.0"	W 74° 30' 18.6"	-	-	-
	St. Sixte	SS-1	N 45° 47' 37.7"	W 75° 12' 02.9"	H	1	B-01
East-Qc	Yamaska	YA-1	N 45° 23' 23.0"	W 72° 27' 01.7"	E, H	3	A-33; A-34; B-02
		YA-2	N 45° 24' 25.0"	W 72° 25' 18.8"	E	-	-
	Chaudière	CH-1	N 45° 22' 48.9"	W 70° 50' 02.9"	H, N	1	A-05
		CH-2	N 45° 29' 03.7"	W 71° 04' 48.3"	E	-	-
	Connecticut	CO-1	N 45° 09' 15.1"	W 71° 32' 53.3"	E, H	4	A-06; A-07; A-08; A-09
		CO-2	N 45° 09' 04.4"	W 71° 32' 48.6"	E, H	3	A-06; A-07; A-09
		CO-3	N 45° 10' 27.2"	W 71° 29' 46.5"	E	-	-
			N 45° 08' 25.9"	W 71° 28' 06.2"	-	-	-
			N 45° 08' 40.2"	W 71° 32' 54.2"	-	-	-
	Richelieu	RI-1	N 45° 15' 03.5"	W 72° 18' 20.8"	H	1	A-10
		RI-2	N 45° 02' 35.8"	W 72° 21' 43.1"	E, H	2	A-11; B-03
		RI-3	N 45° 04' 09.0"	W 72° 21' 11.9"	E, H	2	A-11; B-03
		RI-4	N 45° 03' 01.4"	W 72° 19' 03.3"	E, H	3	A-11; A-12; B-03
			N 45° 02' 28.6"	W 72° 22' 03.9"	-	-	-
			N 45° 06' 31.9"	W 72° 21' 37.0"	-	-	-
			N 45° 01' 33.0"	W 72° 25' 44.5"	-	-	-
			N 45° 01' 47.4"	W 72° 25' 34.6"	-	-	-
			N 45° 06' 31.2"	W 72° 23' 20.9"	-	-	-
			N 45° 01' 52.8"	W 72° 26' 46.5"	-	-	-
			N 45° 16' 05.2"	W 72° 19' 58.0"	-	-	-
			N 45° 06' 02.6"	W 72° 21' 46.3"	-	-	-
			N 45° 01' 46.6"	W 72° 18' 11.7"	-	-	-
			N 45° 10' 02.1"	W 72° 25' 49.1"	-	-	-
	Saint-François	SF-1	N 45° 27' 00.1"	W 71° 49' 32.1"	E, H	6	A-09; A-13; A-14; A-15; A-16; B-04
		SF-2	N 45° 21' 17.1"	W 72° 13' 05.5"	E, H	2	A-26; A-27
		SF-3	N 45° 03' 05.8"	W 72° 11' 39.0"	H	1	B-05
		SF-4	N 45° 14' 01.8"	W 71° 54' 28.0"	E, H	1	B-06
		SF-5	N 45° 13' 30.4"	W 71° 54' 33.6"	E, H	2	B-06; B-07
		SF-6	N 45° 13' 20.6"	W 71° 55' 01.3"	E	-	-
		SF-7	N 45° 12' 56.7"	W 71° 54' 31.5"	E, H	1	B-06
		SF-8	N 45° 13' 58.5"	W 71° 54' 19.4"	E, H	2	B-06; B-07
		SF-9	N 45° 12' 23.0"	W 71° 56' 33.4"	E	-	-
		SF-10	N 45° 12' 37.3"	W 71° 56' 10.2"	E	-	-
		SF-11	N 45° 04' 23.4"	W 71° 52' 37.9"	H	1	A-17
		SF-12	N 45° 07' 48.5"	W 71° 40' 21.9"	E, H, N	2	A-18; A-19
		SF-13	N 45° 10' 36.7"	W 71° 32' 09.5"	E, H	2	A-06; A-07
		SF-14	N 45° 11' 04.5"	W 71° 33' 13.2"	E, H	4	A-06; A-07; A-08; A-09
		SF-15	N 45° 24' 53.2"	W 71° 46' 53.4"	H	3	A-20; A-21; A-22
		SF-16	N 45° 42' 10.6"	W 71° 09' 59.9"	H	2	A-23; A-24
		SF-17	N 45° 38' 15.4"	W 71° 02' 53.8"	H	1	A-24

Region	Drainage Bassin	Sites	Latitude	Longitude	Biotype	No. of hybrid lineages	Names of hybrid lineages
		SF-18	N 45° 03' 45.8"	W 72° 11' 49.5"	H	1	B-05
		SF-19	N 45° 32' 15.2"	W 71° 49' 40.5"	H	2	A-25; B-02
		SF-20	N 45° 25' 36.0"	W 71° 39' 40.9"	E, H	4	A-15; A-29; A-30; A-32
		SF-21	N 45° 25' 50.1"	W 71° 40' 19.4"	E, H	5	A-15; A-28; A-29; A-31; A-32
		SF-22	N 45° 32' 12.9"	W 71° 47' 55.6"	H	1	B-02
		SF-23	N 45° 30' 30.3"	W 71° 58' 48.4"	E	-	-
			N 45° 21' 43.2"	W 71° 49' 44.5"	-	-	-
			N 45° 14' 46.8"	W 71° 55' 08.7"	-	-	-
			N 45° 06' 15.3"	W 71° 39' 35.9"	-	-	-
			N 45° 31' 45.4"	W 71° 15' 50.3"	-	-	-
			N 45° 11' 33.6"	W 72° 19' 30.2"	-	-	-
			N 45° 10' 58.7"	W 71° 58' 13.5"	-	-	-
			N 45° 03' 47.6"	W 71° 53' 16.9"	-	-	-
			N 45° 44' 04.4"	W 71° 07' 46.9"	-	-	-
			N 45° 06' 15.8"	W 71° 38' 54.2"	-	-	-
			N 45° 45' 54.8"	W 71° 05' 50.1"	-	-	-
			N 45° 14' 16.1"	W 71° 51' 58.6"	-	-	-
			N 45° 11' 18.2"	W 71° 33' 60.0"	-	-	-
			N 45° 11' 06.0"	W 72° 18' 30.3"	-	-	-
			N 45° 10' 39.6"	W 71° 52' 53.0"	-	-	-
			N 45° 10' 27.9"	W 71° 53' 13.2"	-	-	-
			N 45° 11' 03.3"	W 71° 32' 36.2"	-	-	-
			N 45° 05' 40.2"	W 72° 18' 27.9"	-	-	-
			N 45° 13' 58.6"	W 71° 52' 55.4"	-	-	-
			N 45° 07' 45.4"	W 72° 13' 56.3"	-	-	-
			N 45° 13' 32.3"	W 71° 32' 36.8"	-	-	-
			N 45° 11' 12.0"	W 71° 51' 06.8"	-	-	-
			N 45° 27' 17.6"	W 71° 55' 10.8"	-	-	-
			N 45° 37' 59.5"	W 71° 03' 06.7"	-	-	-
