# Supplementary material for: Inconsistent phylogeographic pattern between a sperm dependent fish and its host: in situ hybridization vs dispersal
Source: BMC Evol Biol. 2016 Sep 6;16:183. doi: 10.1186/s12862-016-0754-5 (PMC5012089; doi:10.1186/s12862-016-0754-5)
Supplement: Additional file 2: — Multiloci genotype of the parental species C. eos individuals. Nuclear genotype of every C. eos individuals used to assess the paternal species genetic diversity and structure. For each individual, region and sampled sites are provided. (PDF 348 kb) [file 12862_2016_754_MOESM2_ESM.pdf]

Additional file 3. Multiloci genotype of the parental species *C. eos* individuals

Region	Site	Individuals	Microsatellite loci												PEG1/MEST	
			Pho-1		Pho-2		Pho-60		Pho-61		Ca-12		Seat-412			
West-Qc	AS-1	eos-001	282	294	184	216	156	192	154	162	205	213	216	228	222	222
	AS-1	eos-002	250	302	228	244	136	156	154	166	233	253	220	228	222	222
	AS-1	eos-003	250	282	212	248	184	192	166	170	233	233	208	232	222	222
	AS-1	eos-004	250	290	196	244	136	192	154	166	205	213	212	232	222	222
	AS-1	eos-005	282	302	216	224	136	148	154	166	233	233	212	220	222	222
	AS-1	eos-006	282	282	216	236	136	172	142	154	249	253	220	224	222	222
	AS-1	eos-007	250	306	204	236	136	136	142	166	201	213	220	220	222	222
	AS-1	eos-008	282	302	216	236	136	192	154	154	205	245	220	228	222	222
	AS-1	eos-009	254	290	224	240	136	172	166	166	249	253	220	224	222	222
	AS-1	eos-010	302	306	216	240	136	156	142	154	245	253	212	224	222	222
	AS-1	eos-011	302	302	216	220	136	192	154	166	213	245	212	224	222	222
	AS-1	eos-012	302	354	216	220	184	192	166	166	233	245	212	220	222	222
	AS-1	eos-013	286	298	220	244	136	136	166	166	201	233	212	220	222	222
	AS-1	eos-014	298	302	240	260	136	156	154	166	213	233	212	212	222	222
	AS-1	eos-015	254	282	240	240	136	136	154	174	201	253	212	216	222	222
	AS-1	eos-016	282	302	244	248	184	184	166	166	213	233	220	220	222	222
	AS-16	eos-017	266	298	208	232	188	188	154	222	209	245	224	280	222	222
	AS-16	eos-018	NULL	NULL	212	220	172	196	162	190	221	233	220	224	222	222
	AS-16	eos-019	274	298	220	232	192	204	142	178	209	233	228	232	222	222
	AS-16	eos-020	258	266	224	232	184	188	174	206	205	237	200	228	222	222
	AS-16	eos-021	286	298	220	220	204	220	170	174	209	229	212	216	222	222
	AS-16	eos-022	266	278	224	228	172	188	170	202	221	245	224	224	222	222
	AS-16	eos-023	282	294	224	228	200	204	178	254	221	225	212	232	222	222
	AS-16	eos-024	282	294	208	212	180	184	154	170	209	221	216	220	222	222
	AS-16	eos-025	230	274	224	232	160	188	222	242	217	257	224	232	222	222
	AS-16	eos-026	NULL	NULL	212	216	NULL	NULL	146	170	225	269	212	220	222	222
	AS-16	eos-027	262	274	224	224	152	184	138	166	209	225	216	232	222	222
	AS-16	eos-028	262	298	204	204	200	208	158	178	217	225	212	212	222	222
	AS-17	eos-029	262	262	236	236	188	192	150	154	217	241	220	236	222	222
	AS-17	eos-030	258	266	204	208	144	192	154	198	213	217	220	240	227	227
	AS-17	eos-031	318	330	220	220	144	144	182	194	229	241	220	240	227	227
	AS-17	eos-032	314	330	240	240	144	196	182	194	205	215	224	224	227	227
	AS-17	eos-033	258	258	220	220	184	188	182	182	241	241	224	244	227	227
	AS-17	eos-034	258	262	228	228	144	192	154	182	241	241	220	240	222	222
	AS-17	eos-035	258	282	240	240	184	188	198	218	213	217	216	240	227	227
	AS-17	eos-036	258	282	220	240	184	188	154	182	241	241	220	232	227	227
	AS-17	eos-037	258	270	228	228	188	192	154	170	213	241	220	228	227	227
	AS-17	eos-038	302	326	228	228	144	196	154	182	205	241	216	220	227	227
	AS-17	eos-039	250	250	228	228	152	188	146	150	215	241	220	236	227	227
	AS-17	eos-040	254	302	228	228	192	196	154	154	213	241	224	240	222	222
	AS-17	eos-041	302	338	192	228	144	196	154	154	241	245	220	224	227	227
	AS-17	eos-042	262	262	208	208	184	192	158	186	213	241	220	240	227	227
	AS-17	eos-043	262	322	228	240	NULL	NULL	154	198	241	241	220	232	227	227
	AS-17	eos-044	262	262	240	248	196	200	150	182	241	241	220	240	227	227
	AS-17	eos-045	242	326	188	228	184	184	182	198	241	241	224	240	227	227
	AS-17	eos-046	258	266	188	240	180	184	150	178	205	241	220	244	222	227
	AS-17	eos-047	262	314	228	228	184	184	154	194	241	241	220	240	222	227
	AS-17	eos-048	262	302	240	240	144	196	178	182	215	241	224	240	222	227
	AS-17	eos-049	262	266	228	240	144	192	154	182	213	241	232	240	227	227
	AS-17	eos-050	262	302	232	232	184	196	182	222	241	241	224	236	227	227

Region	Site	Individuals	Microsatellite loci												PEG1/MEST	
			Pho-1		Pho-2		Pho-60		Pho-61		Ca-12		Seat-412			
	AS-17	eos-051	262	338	NULL	NULL	144	144	154	154	NULL	NULL	NULL	NULL	227	227
	AS-17	eos-052	326	342	NULL	NULL	144	196	154	190	205	241	NULL	NULL	222	227
	AS-5	eos-053	258	330	176	228	176	176	162	190	225	225	220	228	222	222
	AS-5	eos-054	258	270	188	200	152	196	154	202	217	229	208	228	222	222
	AS-5	eos-055	234	254	220	232	148	216	NULL	NULL	221	225	220	248	222	222
	AS-5	eos-056	234	266	196	232	144	200	182	206	229	245	220	228	222	222
	AS-5	eos-057	262	262	216	224	144	188	158	194	221	221	204	208	222	222
	AS-5	eos-058	262	262	200	224	140	140	194	206	225	245	220	220	222	222
	AS-5	eos-059	262	262	192	196	144	200	198	214	225	233	204	228	222	222
	AS-5	eos-060	242	254	216	224	200	204	158	186	233	233	216	220	222	222
	AS-5	eos-061	262	266	220	224	200	200	170	186	217	221	216	232	222	222
	AS-5	eos-062	258	270	224	232	140	144	146	154	217	225	200	216	222	222
	AS-5	eos-063	278	282	196	212	176	184	194	194	213	241	212	232	222	222
	AS-5	eos-064	270	286	216	228	140	184	162	210	233	237	192	192	222	222
	AS-5	eos-065	282	306	200	232	152	152	NULL	NULL	NULL	NULL	208	212	222	222
	AS-5	eos-066	270	278	200	200	140	200	154	154	205	217	216	224	222	222
	AS-5	eos-067	258	278	196	212	140	144	142	206	209	229	216	220	222	222
	AS-5	eos-068	270	282	196	208	140	144	150	162	205	229	212	236	222	222
	AS-5	eos-069	254	262	216	224	136	188	150	158	225	241	216	224	222	222
	AS-5	eos-070	258	270	208	216	144	144	174	174	225	241	212	216	222	222
	AS-5	eos-071	262	274	248	260	188	188	138	142	217	237	212	236	222	222
	AS-5	eos-072	278	282	NULL	NULL	144	204	NULL	NULL	NULL	NULL	NULL	NULL	222	222
	AS-5	eos-073	274	286	212	212	140	148	150	154	221	237	212	220	222	222
	AS-5	eos-074	262	262	224	228	136	200	NULL	NULL	213	221	212	224	222	222
	AS-5	eos-075	262	302	228	232	140	192	154	154	217	233	204	216	222	222
	AS-5	eos-076	262	270	196	196	148	156	138	146	217	217	212	232	222	222
	AS-5	eos-077	258	266	224	228	152	180	NULL	NULL	209	221	NULL	NULL	222	222
	BA-1	eos-078	258	266	220	220	144	148	154	162	201	201	196	252	222	222
	BA-1	eos-079	258	270	110	110	144	156	154	162	197	237	196	200	222	222
	BA-1	eos-080	254	274	192	196	168	172	174	174	225	229	208	220	222	222
	BA-1	eos-081	250	278	110	232	156	160	150	150	225	233	196	204	222	222
	BA-1	eos-082	282	294	110	110	148	148	150	150	201	249	200	212	222	222
	BA-1	eos-083	282	302	110	212	152	168	150	162	229	237	220	232	222	222
	BA-1	eos-084	286	298	NULL	NULL	120	144	NULL	NULL	NULL	NULL	NULL	NULL	222	222
	BA-1	eos-085	282	294	196	212	152	164	146	146	237	245	192	248	222	222
	BA-1	eos-086	258	302	114	184	136	192	146	158	229	237	196	204	222	222
	BA-1	eos-087	266	286	180	184	144	148	154	178	205	257	200	256	222	222
	BA-1	eos-088	262	278	110	200	156	192	146	154	233	237	212	252	222	222
	BA-1	eos-089	254	278	188	188	148	156	166	166	201	241	208	220	222	222
	BA-1	eos-090	262	298	180	216	148	184	150	170	221	233	200	200	222	222
	BA-1	eos-091	234	286	204	204	144	160	142	158	217	221	204	232	222	222
	BA-1	eos-092	282	294	110	110	152	168	158	158	225	241	204	216	222	222
	BA-1	eos-093	250	250	110	110	144	148	146	154	229	237	204	216	222	222
	BA-1	eos-094	294	298	200	200	132	152	154	158	213	249	196	216	222	222
	BA-1	eos-095	290	290	204	204	156	168	154	178	201	229	188	212	222	222
	BA-1	eos-096	250	258	NULL	NULL	160	192	142	146	213	233	196	208	222	222
	BA-1	eos-097	278	306	204	204	144	148	150	158	241	241	188	248	222	222
	BA-1	eos-098	266	282	110	208	136	172	162	162	205	229	204	212	222	222
	BA-1	eos-099	290	294	188	208	148	180	114	158	201	233	208	216	222	222
	BA-1	eos-100	286	298	110	110	156	156	146	174	217	221	200	208	222	222
	BA-1	eos-101	282	294	NULL	NULL	144	160	146	158	217	233	220	228	222	222
	BA-1	eos-102	238	258	NULL	NULL	144	172	150	174	229	237	188	216	222	222

Region	Site	Individuals	Microsatellite loci												PEG1/MEST	
			Pho-1		Pho-2		Pho-60		Pho-61		Ca-12		Seat-412			
	NO-10	eos-103	290	302	216	216	160	184	NULL	NULL	233	233	220	232	222	222
	NO-10	eos-104	NULL	NULL	216	216	192	208	NULL	NULL	245	253	212	220	222	222
	NO-10	eos-105	282	286	216	216	192	200	NULL	NULL	245	253	212	224	222	222
	NO-10	eos-106	282	318	236	240	148	156	150	154	229	245	220	220	222	222
	NO-10	eos-107	274	282	216	216	136	184	150	154	233	245	220	224	222	222
	NO-10	eos-108	282	302	232	236	196	204	166	170	213	233	220	232	222	222
	NO-10	eos-109	254	302	220	220	164	172	166	174	217	253	212	220	222	222
	NO-10	eos-110	274	302	216	216	136	192	154	166	233	245	220	224	222	222
	NO-10	eos-111	282	302	212	216	136	204	150	154	209	249	224	224	222	222
	NO-10	eos-112	274	282	216	220	168	196	154	162	237	237	208	220	222	222
	NO-10	eos-113	254	274	216	236	148	164	154	178	213	253	216	224	227	227
	NO-10	eos-114	274	282	216	240	136	160	162	174	205	213	216	224	222	222
	NO-10	eos-115	254	274	216	240	192	204	154	154	249	253	220	224	222	222
	NO-10	eos-116	282	290	216	216	196	228	162	166	205	253	224	224	222	222
	NO-10	eos-117	286	302	216	236	168	192	170	174	213	253	220	224	222	222
	NO-10	eos-118	254	282	228	236	184	204	150	154	225	233	220	224	222	222
	NO-10	eos-119	254	286	216	216	136	192	150	166	213	245	220	220	222	222
	NO-10	eos-120	282	298	200	240	148	192	154	166	233	253	216	232	222	222
	NO-10	eos-121	226	282	216	244	NULL	NULL	146	166	217	253	220	220	222	222
	RO-1	eos-122	266	282	216	240	152	156	146	146	201	225	228	240	227	227
	RO-1	eos-123	278	314	236	236	136	152	154	158	201	203	212	220	222	227
	RO-1	eos-124	278	286	212	232	144	160	170	170	205	217	216	220	227	227
	RO-1	eos-125	262	278	224	224	132	136	NULL	NULL	201	205	224	244	227	227
	RO-1	eos-126	254	270	252	252	168	168	154	170	203	209	212	228	227	227
	RO-1	eos-127	266	270	220	228	152	152	150	150	201	221	200	228	227	227
	RO-1	eos-128	270	274	212	228	148	168	150	158	201	203	224	236	227	227
	RO-1	eos-129	274	286	236	244	144	164	154	170	209	229	216	228	222	227
	RO-1	eos-130	282	282	248	248	140	152	178	178	203	205	216	232	227	227
	RO-1	eos-131	254	274	212	232	148	184	166	202	191	199	224	232	227	227
	RO-1	eos-132	266	282	200	224	144	160	NULL	NULL	209	209	224	236	222	227
East-Qc	YA-1	eos-133	242	290	110	288	140	144	154	158	241	245	216	220	222	222
	YA-1	eos-134	250	254	192	276	144	164	162	174	221	241	204	212	222	222
	YA-1	eos-135	286	310	208	208	152	152	162	170	217	225	204	208	222	222
	YA-1	eos-136	258	306	224	228	156	156	166	174	237	245	208	232	222	222
	YA-1	eos-137	278	278	110	116	136	168	138	170	213	233	204	232	222	222
	YA-1	eos-138	282	298	NULL	NULL	164	172	162	162	219	221	204	224	222	222
	YA-1	eos-139	298	326	110	192	148	152	158	158	219	245	208	208	222	222
	YA-1	eos-140	242	318	110	220	144	156	162	162	203	245	208	208	222	222
	YA-1	eos-141	246	254	204	204	144	148	158	158	217	221	NULL	NULL	222	222
	YA-1	eos-142	258	278	110	200	156	164	174	178	217	229	204	208	222	222
	YA-1	eos-143	270	314	184	184	156	164	154	170	213	229	204	220	222	222
	YA-1	eos-144	266	282	196	196	156	160	158	166	201	229	212	232	222	222
	YA-1	eos-145	262	286	110	228	152	160	154	166	209	221	216	232	222	222
	YA-1	eos-146	258	270	180	216	152	156	138	174	241	249	196	204	222	222
	YA-1	eos-147	290	306	110	184	160	160	174	178	237	245	202	204	222	222
	YA-1	eos-148	258	326	110	180	152	156	174	222	221	241	200	220	222	222
	YA-1	eos-149	250	298	110	232	156	156	154	162	217	217	208	216	222	222
	YA-1	eos-150	258	310	208	208	156	156	NULL	NULL	229	241	192	232	222	222
	CO-1	eos-151	306	306	212	220	140	160	162	182	221	221	216	236	222	222
	CO-1	eos-152	290	306	200	212	152	160	166	166	225	225	200	216	222	222
	CO-1	eos-153	286	306	176	228	148	160	148	166	225	225	208	284	222	222
	CO-1	eos-154	302	310	192	236	140	156	150	162	217	225	224	228	222	222

Region	Site	Individuals	Microsatellite loci												PEG1/MEST	
			Pho-1		Pho-2		Pho-60		Pho-61		Ca-12		Seat-412			
	CO-1	eos-155	310	310	244	244	156	160	158	166	209	229	236	280	222	227
	CO-1	eos-156	306	310	110	212	140	184	150	158	205	217	216	280	222	227
	CO-1	eos-157	NULL	NULL	208	216	152	152	166	170	225	225	188	204	222	222
	CO-1	eos-158	286	294	176	208	140	152	162	166	209	221	192	204	222	222
	RI-2	eos-159	282	310	212	236	148	180	158	170	209	225	240	240	222	222
	RI-2	eos-160	270	282	224	228	148	176	154	158	229	253	204	236	222	222
	RI-2	eos-161	258	278	220	220	152	156	154	174	197	241	208	208	222	222
	RI-2	eos-162	246	322	212	216	152	164	182	182	201	225	204	212	222	222
	RI-2	eos-163	270	274	184	192	152	156	154	158	201	261	204	212	222	222
	RI-2	eos-164	294	322	188	216	152	164	NULL	NULL	209	225	208	212	222	222
	RI-2	eos-165	258	286	212	216	160	164	154	162	201	245	200	212	222	222
	RI-2	eos-166	290	290	236	236	144	148	NULL	NULL	225	245	216	220	222	222
	RI-2	eos-167	274	274	184	196	148	156	154	166	225	233	196	224	222	222
	RI-2	eos-168	266	274	228	228	148	160	146	170	201	205	212	212	222	222
	RI-2	eos-169	266	266	224	228	144	156	170	186	221	241	196	220	222	222
	RI-2	eos-170	278	322	180	224	148	160	158	166	229	241	212	212	222	222
	RI-2	eos-171	286	290	184	240	148	152	138	170	225	225	212	212	222	222
	RI-2	eos-172	250	274	184	220	176	180	150	166	209	233	212	220	222	227
	RI-4	eos-173	266	278	180	192	144	152	186	186	197	249	212	228	222	222
	RI-4	eos-174	242	278	224	232	144	152	166	166	225	237	192	232	222	227
	RI-4	eos-175	262	278	184	212	152	152	146	174	241	257	200	228	222	227
	RI-4	eos-176	282	282	212	212	152	152	170	174	225	241	216	228	222	222
	RI-4	eos-177	270	270	212	216	144	180	162	190	221	237	200	204	222	227
	RI-4	eos-178	306	322	184	188	148	148	154	174	241	245	212	236	222	227
	RI-4	eos-179	262	322	208	212	140	156	138	166	237	245	208	228	222	227
	RI-4	eos-180	274	274	200	224	160	164	146	166	245	269	212	216	227	227
	SF-2	eos-181	270	282	110	110	164	164	158	166	225	233	220	232	222	222
	SF-2	eos-182	282	326	232	236	168	220	154	170	213	237	208	220	222	222
	SF-2	eos-183	270	314	208	212	168	172	138	146	201	205	208	212	222	227
	SF-2	eos-184	262	294	228	232	144	164	150	150	205	213	212	220	222	222
	SF-2	eos-185	286	286	172	172	152	156	154	186	229	241	200	212	222	222
	SF-2	eos-186	266	306	208	224	144	148	142	166	213	217	200	216	222	227
	SF-2	eos-187	266	282	208	212	164	196	146	146	229	233	204	224	222	222
	SF-2	eos-188	294	322	212	236	148	192	146	186	197	205	208	220	222	222
	SF-2	eos-189	250	314	216	224	156	164	NULL	NULL	201	209	NULL	NULL	222	222
	SF-2	eos-190	282	302	110	216	160	172	162	174	221	237	200	268	222	227
	SF-2	eos-191	262	266	110	224	160	168	162	166	209	229	204	208	222	227
	SF-4	eos-192	234	282	212	220	152	156	166	178	221	225	200	220	227	227
	SF-4	eos-193	258	298	110	240	160	172	162	178	217	221	212	216	227	227
	SF-4	eos-194	258	266	110	204	140	216	138	166	213	241	216	280	222	222
	SF-4	eos-195	270	302	196	216	144	204	170	174	209	233	208	268	222	222
	SF-4	eos-196	254	286	110	212	140	160	162	162	217	237	208	226	227	227
	SF-4	eos-197	286	298	196	200	140	148	NULL	NULL	209	249	NULL	NULL	227	227
	SF-4	eos-198	234	266	212	212	148	152	NULL	NULL	209	233	216	264	227	227
	SF-4	eos-199	274	298	192	276	156	156	NULL	NULL	213	233	NULL	NULL	227	227
	SF-4	eos-200	262	334	204	244	172	200	178	186	209	233	204	264	227	227
	SF-4	eos-201	246	270	188	188	140	148	170	178	229	241	216	216	227	227
	SF-4	eos-202	274	298	208	248	156	168	142	142	221	241	208	216	227	227
	SF-4	eos-203	246	262	212	212	148	200	146	158	209	237	224	268	227	227
	SF-4	eos-204	254	262	196	196	136	164	146	162	229	245	216	220	222	222
	SF-4	eos-205	258	262	196	204	212	216	126	162	217	221	208	208	222	227
	SF-4	eos-206	274	278	200	208	168	200	166	170	225	241	220	264	227	227

Region	Site	Individuals	Microsatellite loci												PEG1/MEST	
			Pho-1		Pho-2		Pho-60		Pho-61		Ca-12		Seat-412			
	SF-4	eos-207	258	298	200	272	152	156	166	182	221	241	204	272	222	222
	SF-4	eos-208	290	302	212	244	140	196	134	170	225	241	212	268	227	227
	SF-10	eos-209	306	306	196	228	140	144	150	170	209	225	212	220	222	227
	SF-10	eos-210	266	306	176	236	140	148	158	166	209	209	216	216	222	227
	SF-10	eos-211	278	298	114	216	140	148	NULL	NULL	209	209	220	220	227	227
	SF-10	eos-212	266	266	196	216	148	188	NULL	NULL	209	221	216	228	227	227
	SF-10	eos-213	278	306	114	216	140	152	150	194	209	233	200	220	227	227
	SF-10	eos-214	190	306	196	216	148	164	166	166	209	233	216	220	227	227
	SF-10	eos-215	298	306	188	216	164	164	150	194	221	221	292	292	227	227
	SF-10	eos-216	190	290	110	228	140	164	150	166	209	221	216	216	227	227
	SF-10	eos-217	190	266	110	196	148	156	166	166	209	221	200	216	222	227
	SF-10	eos-218	266	306	196	196	148	156	166	170	209	209	200	220	227	227
	SF-12	eos-219	310	318	110	224	152	196	154	162	209	213	212	236	227	227
	SF-12	eos-220	290	294	196	224	152	156	150	154	209	233	212	216	227	227
	SF-12	eos-221	246	314	192	192	148	200	170	182	209	233	216	224	227	227
	SF-12	eos-222	310	330	196	228	156	192	154	170	229	229	212	224	227	227
	SF-12	eos-223	226	238	228	228	148	152	NULL	NULL	209	217	NULL	NULL	227	227
	SF-12	eos-224	290	290	204	240	148	160	NULL	NULL	225	241	208	216	222	227
	SF-12	eos-225	238	302	110	204	152	192	NULL	NULL	217	245	212	216	222	227
	SF-12	eos-226	294	294	240	240	160	192	150	150	229	229	212	216	222	227
	SF-12	eos-227	294	318	110	216	152	152	162	178	225	229	220	228	222	227
	SF-12	eos-228	282	290	200	200	152	196	170	170	201	233	204	216	222	222
	SF-12	eos-229	302	322	192	240	156	196	146	158	221	225	224	228	227	227
	SF-12	eos-230	318	318	220	264	128	152	138	154	209	225	224	224	222	222
	SF-12	eos-231	238	298	114	212	148	192	142	158	213	221	208	224	227	227
	SF-12	eos-232	290	310	110	216	140	148	154	162	213	225	196	220	222	222
	SF-12	eos-233	282	330	200	204	140	156	166	166	237	245	232	252	222	222
	SF-12	eos-234	294	314	110	220	148	188	142	166	213	237	228	252	227	227
	SF-12	eos-235	294	294	212	216	196	196	NULL	NULL	229	313	NULL	NULL	227	227
	SF-14	eos-236	NULL	NULL	196	224	144	164	142	166	213	213	208	216	222	222
	SF-14	eos-237	282	314	220	234	148	160	162	170	209	219	232	280	222	222
	SF-14	eos-238	298	306	192	216	184	184	142	170	209	209	212	228	222	227
	SF-14	eos-239	274	282	204	204	152	184	170	174	217	221	224	228	222	227
	SF-14	eos-240	302	310	228	228	148	156	154	158	209	229	148	154	222	227
	SF-14	eos-241	230	238	216	260	148	148	154	166	209	217	216	244	222	227
	SF-14	eos-242	294	306	244	244	148	184	134	154	207	207	204	256	222	227
	SF-14	eos-243	266	302	212	212	164	184	154	226	217	221	224	240	222	222
	SF-14	eos-244	270	286	216	216	148	152	162	166	209	221	196	200	222	227
	SF-14	eos-245	302	310	216	220	148	148	150	170	205	225	200	228	222	227
	SF-14	eos-246	274	298	204	212	148	156	146	162	213	217	200	224	222	227
	SF-14	eos-247	278	314	NULL	NULL	140	144	162	166	205	213	200	220	222	222
	SF-14	eos-248	286	310	188	232	144	148	154	178	205	209	216	220	222	227
	SF-14	eos-249	294	298	212	272	152	188	174	198	215	217	228	240	222	227
	SF-14	eos-250	282	298	204	228	152	184	174	198	205	213	220	232	222	227
