# Supplementary material for: Inconsistent phylogeographic pattern between a sperm dependent fish and its host: in situ hybridization vs dispersal
Source: BMC Evol Biol. 2016 Sep 6;16:183. doi: 10.1186/s12862-016-0754-5 (PMC5012089; doi:10.1186/s12862-016-0754-5)
Supplement: Additional file 3: — Consensus nuclear genotype and mitochondrial haplotype of the different lineages. Nuclear genotype and mitochondrial haplotype of the different lineages. Allele size refers to the more common genotype and bold characters to variable loci within a given lineage. (PDF 46 kb) [file 12862_2016_754_MOESM3_ESM.pdf]

Additional file 2. Consensus nuclear genotype and mitochondrial haplotype of the different lineages. Allele size refers to the more common clone and bold characters to variable loci within a given lineage. Lineages marked with an asterisk refer to triploid individuals for which the hybrid genotype was not determined.

Hybrid lineage	Nuclear loci									Sample size
	PEG1/MEST		Pho-1	Pho-2	Pho-60	Pho-61	Ca-12		Seat-412	
	C. eos	*C. neogaeus*					*C. eos*	*C. neogaeus*		
A-01	222	170	270	**204**	**148**	**146**	209	**375**	**210 / 212**	50
A-02	222	170	278	220	152	154	213	245	190 / 258	1
A-03*	222	170	302 / 310	196 / 200	168 / 176	166	249	357	204 / 242	2
A-04*	222 / 227	170	278 / 290	224 / 228	168 / 180	NULL	207 / 217	247	204 / 214 / 234	1
A-05*	227	170	302 / 322	200 / 204	148 / 156	162 / 170	201 / 225	265	222 / 228	1
A-06	222	170	**306**	176	**184**	**162**	205	**281**	**230 / 280**	60
A-07	222	170	**298**	**200**	148	**158**	**209**	**325**	**216 / 226**	28
A-08	227	170	262	**208**	152	**166**	209	**317**	**238 / 264**	12
A-09	222	170	**302**	**236**	152	**158**	**225**	**305**	**214 / 240**	15
A-10	227	170	**278**	**204**	**136**	170	209	241	**174 / 252**	4
A-11	222	170	**326**	**208**	164	**174**	221	**245**	**222 / 242**	27
A-12	222	170	286	208	152	**170**	**233**	**253**	**206** / 222	2
A-13	222	170	**286**	228	156	166	217	317	214 / 228	3
A-14*	222	170	266 / 294	252	156	158 / 178	221 / 229	309	204 / 218	1
A-15	222	170	274	212	160	**146**	221	349	210 / **240**	19
A-16*	222 / 227	170	298 / 306	192 / 204	148 / 156	182 / 186	209	337	204 / 214 / 224	1
A-17*	222	170	326	204/236	152	170	225	321	214 / 216 / 234	1
A-18	222	170	294	212	196	**170**	**229**	**309**	196 / **222**	47
A-19*	222	170	274 / 310	212	152 / 160	148 / 166	217	245	210 / 216 / 240	1
A-20	222	170	**306**	**180**	**188**	**166**	209	277	**222** / 288	3
A-21	222	170	274	216	164	150	225	349	210 / 236	2
A-22*	222	170	278 / 302	212	156 / 164	148 / 154	209	225	208 / 210 / 244	1
A-23	222	170	278	208	164	142	217	337	210 / 240	2
A-24	222	170	310	**260**	148	150	209	**289**	220 / 226	4
A-25*	222	170	278 / 302	204 / 244	144 / 152	174	209 / 211	213	218 / 224	1
A-26*	222	170	262 / 322	204 / 220	160 / 212	158 / 162	225	241	216 / 220 / 238	1
A-27	222	170	262	**228**	148	**146**	**213**	**305**	218 / 220	6
A-28	227	170	286	176	156	142	217	293	234 / 244	1
A-29	222	170	290	172	144	178	209	269	230 / 232	2
A-30*	222	170	282 / 310	180	136 / 184	166	205 / 225	277	216 / 232 / 290	1

Hybrid lineage	Nuclear loci									Sample size
	PEG1/MEST		Pho-1	Pho-2	Pho-60	Pho-61	Ca-12		Seat-412	
	C. eos	*C. neogaeus*					*C. eos*	*C. neogaeus*		
A-31*	222	170	298 / 318	184 / 204	144 / 148	162 / 182	209	329	216 / 218 / 268	1
A-32	222	170	326	204	152	170	225	269	216 / 218	2
A-33*	222	170	254 / 334	216	160 / 164	166 / 176	209	241	198 / 210 / 236	1
A-34	222	170	**306**	**220**	**156**	**174**	**245**	**309**	208 / **234**	15
B-01	222	170	**298**	**212**	160	**174**	**209**	**245**	**220 / 230**	280
B-02	222	170	**250**	112	**172**	162	217	**389**	**208 / 212**	30
B-03	227	170	**274**	**192**	**176**	**150**	**209**	**329**	**204 / 206**	10
B-04*	222 / 227	170	302	244 / 236	160	170	217	**225**	208 / **216**	2
B-05*	222	170	286 / 290	228	164	162	213	225	196 / 244	2
B-06	227	170	**254**	112	160	170	217	**237**	208 / 226	39
B-07	222	170	**266**	**204**	156	170	209	**333**	206 / 208	4
